# Multi-omics analysis revealed the mechanism underlying flavonol biosynthesis during petal color formation in *Camellia Nitidissima*

**DOI:** 10.1186/s12870-024-05332-w

**Published:** 2024-09-09

**Authors:** Yi Feng, Jiyuan Li, Hengfu Yin, Jian Shen, Weixin Liu

**Affiliations:** 1 Key Laboratory of Tree Breeding of Zhejiang Province, Research Institute of Subtropical Forestry, Chinese Academy of Forestry, Hangzhou, Zhejiang 311400 China; 2 Jinhua Forestry Technology Promotion Station of Zhejiang Province, Jinhua, Zhejiang 321017 China

**Keywords:** *Camellia nitidissima*, Flavonol, Multi-omics analysis, *CnFLS2*, Jasmonate

## Abstract

**Background:**

*Camellia nitidissima* is a rare, prized camellia species with golden-yellow flowers. It has a high ornamental, medicinal, and economic value. Previous studies have shown substantial flavonol accumulation in *C. nitidissima* petals during flower formation. However, the mechanisms underlying the golden flower formation in *C. nitidissima* remain largely unknown.

**Results:**

We performed an integrative analysis of the transcriptome, proteome, and metabolome of the petals at five flower developmental stages to construct the regulatory network underlying golden flower formation in *C. nitidissima*. Metabolome analysis revealed the presence of 323 flavonoids, and two flavonols, quercetin glycosides and kaempferol glycosides, were highly accumulated in the golden petals. Transcriptome and proteome sequencing suggested that the flavonol biosynthesis-related genes and proteins upregulated and the anthocyanin and proanthocyanidin biosynthesis-related genes and proteins downregulated in the golden petal stage. Further investigation revealed the involvement of *MYB*s and *bHLHs* in flavonoid biosynthesis. Expression analysis showed that *flavonol synthase 2* (*CnFLS2*) was highly expressed in the petals, and its expression positively correlated with flavonol content at all flower developmental stages. Transient overexpression of *CnFLS2* in the petals increased flavonol content. Furthermore, correlation analysis showed that the jasmonate (JA) pathways positively correlated with flavonol biosynthesis, and exogenous methyl jasmonate (MeJA) treatment promoted *CnFLS2* expression and flavonol accumulation.

**Conclusions:**

Our findings showed that the JA-*CnFLS2* module regulates flavonol biosynthesis during golden petal formation in *C. nitidissima*.

**Supplementary Information:**

The online version contains supplementary material available at 10.1186/s12870-024-05332-w.

## Background

Flavonoids form the largest pigment group in plants and determine the flower color in most plants [1, 2]. Based on their structures, flavonoids can be classified into flavonols, anthocyanins, proanthocyanidins, flavones, isoflavones, chalcones, aurones and so on [3]. Anthocyanins impart red, orange, purple, and blue colors in plants [4]. Flavonols, chalcones, and aurones are the important pale-yellow and yellow pigments [5, 6]. Flavonoids are edible pigments and taste-regulating components of wine and plant-derived foods [7–9]. They also exhibit several health-benefiting properties, such as antioxidant, vasodilator, anti-carcinogenic, and anti-aging [10, 11].

Flavonoids are synthesized via the phenylpropanoid pathway [12]. More than 9000 flavonoids have been identified in plants [3, 13]. Flavonols are an important component of the flavonoid biosynthesis pathway. The biosynthesis of flavonols begins with phenylalanine, which is converted to coumaroyl-CoA under the actions of phenylalanine ammonia lyase (PAL), cinnamic acid 4-hydroxylase (C4H), and 4-coumarate-CoA ligase (4CL) [8, 14]. Coumaroyl-CoA is then converted to dihydroflavonols, including dihydrokaempferol (DHK), dihydroquercetin (DHQ), and dihydromyricetin (DHM), under the catalysis of chalcone synthase (CHS), chalcone isomerase (CHI), flavanone 3-hydroxylase (F3H), flavanone 3′-hydroxylase (F3′H), and flavanone 3′, 5′-hydroxylase (F3′5′H) [15–17]. Dihydroflavonols are the key intermediate metabolites in flavonoid biosynthesis. They are converted to flavonols under the activity of flavonol synthase (FLS) [18]. In addition, dihydroflavonols can be converted to anthocyanin and proanthocyanidin under the catalysis of dihydroflavonol 4-reductase (DFR), leucoanthocyanidin reductase (LAR), anthocyanidin synthase (ANS), and anthocyanidin reductase (ANR) [19]. DFR and FLS compete for the substrate dihydroflavonols, diverting them towards either anthocyanin and proanthocyanidin biosynthesis or flavonol biosynthesis, respectively [20].

MYB transcription factors and their MBW complex, comprising MYB, bHLH, and WD40, are the most widely studied factors involved in the transcriptional regulation of flavonol metabolism [21, 22]. In *Arabidopsis thaliana*, subgroup 7 MYB family members AtMYB11, AtMYB12, and AtMYB111 activate *CHS*, *CHI*, *F3H*, and *FLS* genes, increasing flavonol levels [23]. In *Cucumis sativus* CsMYB60, an AtMYB111 homolog, activates *CsFLS* and *CsLAR* by binding to their promoters, leading to *Cs4CL* and *CsCHS* upregulation and enhanced biosynthesis of flavonols and proanthocyanidins [24]. In addition to MYB transcription factors, several hormones, such as jasmonate (JA), auxin, ethylene, and gibberellic acid (GA), are involved in the regulation of flavonol metabolism in plants. For instance, preharvest methyl jasmonate (MeJA) treatment has been shown to increase the flavonol content in red raspberry [25] and promote anthocyanin accumulation in *Arabidopsis* [26].

There are more than 20,000 *Camellia* species and varieties. Most of them are red flower varieties, with rare yellow flower varieties (accounting for < 1% of all varieties) [27, 28]. *Camellia nitidissima* is a rare and prized species of the genus *Camellia*. It is known for its unique golden flower color, referred to as “giant panda in the plant world” and “the queen of camellias,” and listed as a national second-class protected plant in China [29]. It has a high ornamental and economic value. The golden flowers of *C. nitidissima* are rich in flavonoids, especially flavonols [30–32]. These components exhibit antioxidant, anti-aging, lipid-lowering, and blood pressure-lowering effects and have a huge economic value in medical care and food production [10, 11]. Therefore, *C. nitidissima* is a precious species, integrating ornamental, medicinal, and edible functions. In addition, it is a valuable resource for research on yellow camellia flower formation and the breeding of yellow camellia varieties.

Previous studies have shown that flavonols were the main pigments present in the golden petals of *C. nitidissima* [30, 31]. However, the mechanisms underlying flavonol biosynthesis during golden color formation in *C. nitidissima* petals remain largely unknown. In the present study, we assessed the petals of *C. nitidissima* at five different flower developmental stages and used several approaches, including multi-omics analyses; evaluation of the metabolome, transcriptome, and proteome; transient transfection; and MeJA treatment, to determine the potential pathways underlying flavonol accumulation during golden flower formation in *C. nitidissima*.

## Results

### Metabolome analysis of *C. nitidissima* petals

**Fig. 1:**
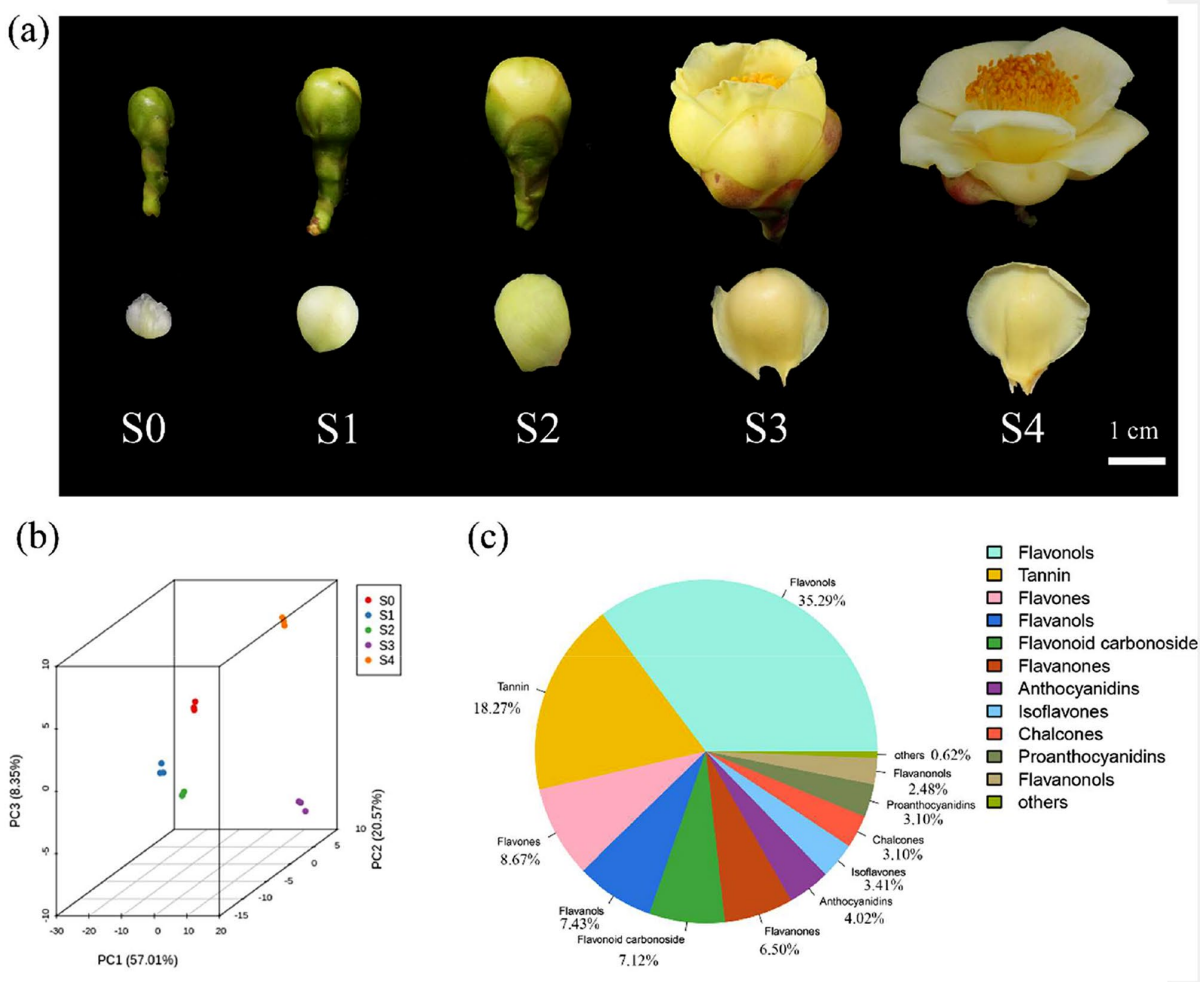
Petals development and metabolome analysis. (**a**) Five developmental stages of petals in *C. nitidissima.* S0, early-bud stage; S1, mid-bud stage; S2, late-bud stage; S3, half-opening stage; S4, complete-opening stage. (**b**) OPLS-DA Score Map. Each point in the graph indicates a sample, and samples of the same group are represented with the same color. (**c**) Number of differential metabolites

We observed the phenotypic changes in petals during the five developmental stages of the flowers (Fig. 1a). We used UPLC-ESI-MS/MS to analyze the changes in flavonoid metabolism in the petals. We obtained 15 petal samples and divided them into five groups with three samples each. The OPLS-DA score map showed good variability among the groups (Fig. 1b). A total of 323 flavonoids were screened, including 114 flavonols (35.29%), 59 tannins (18.27%), 28 flavones (8.67%), 24 flavanols (7.43%), 23 flavonoid carbonosides (7.12%), 21 flavanones (6.50%), 13 anthocyanins (4.02%), 11 isoflavones (3.41%), 10 proanthocyanidins (3.10%), 10 chalcones (3.10%), and 8 flavanonols (2.48%) (Fig. 1c).

### Analysis of differentially expressed metabolites (DEMs)

**Fig. 2:**
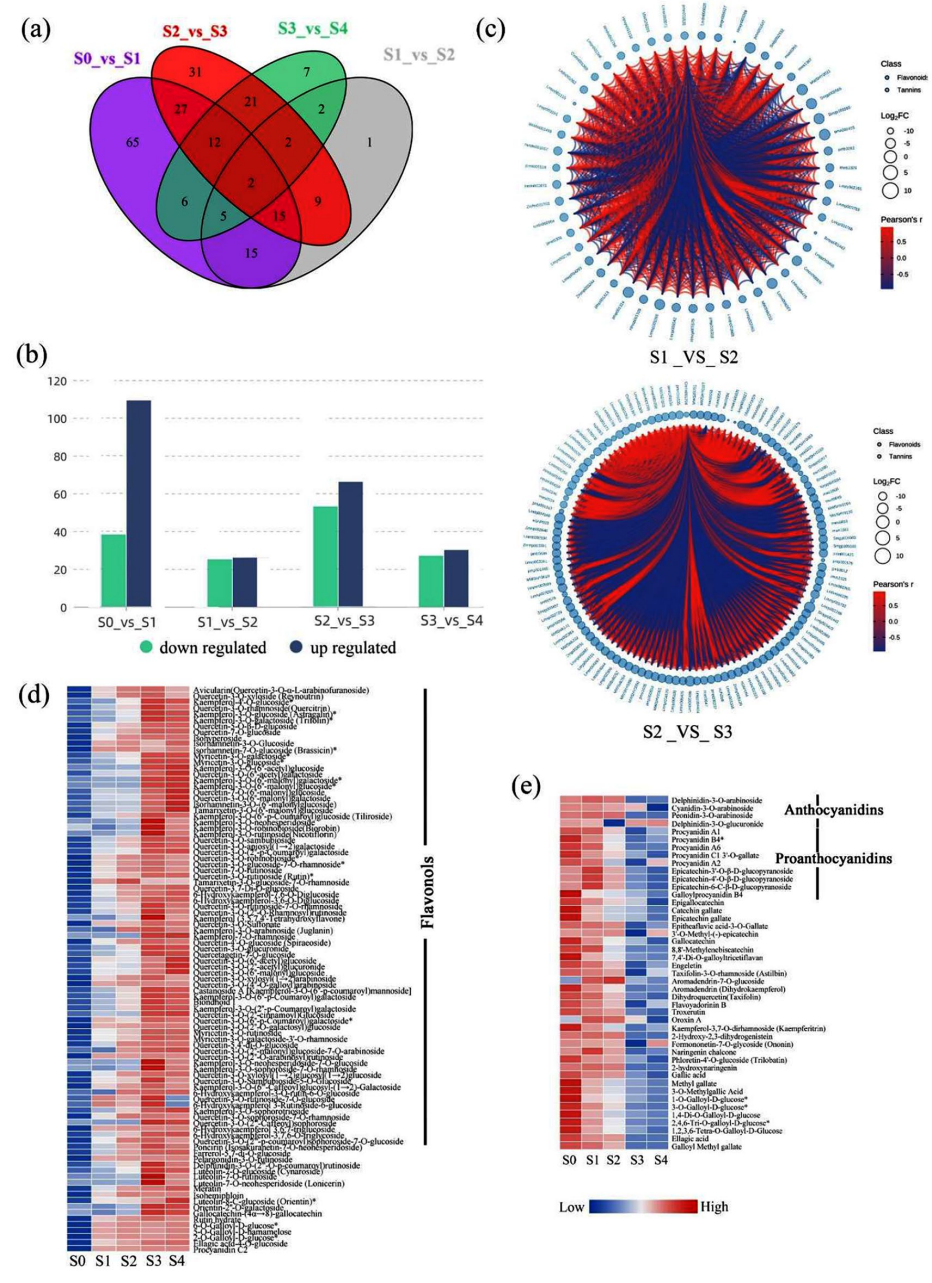
Differentially expressed metabolites (DEMs) analysis. (**a**) The Venn diagram depicts the common and unique number of (shared and unique) DEMs among the four groups of samples, respectively. (**b**) Up- and downregulated unigenes in different comparisons. The horizontal coordinate represents the number of DEMs, and the vertical coordinate represents different flowering periods. (**c**) The Pearson correlation analysis between comparisons. The red line represents positive correlation, and the blue line represents negative correlation. Heat maps of positively (**d**) and negatively (**e**) correlated metabolites during the formation of golden flowers. Blue indicates low expression and red indicates high expression

A total of 285 DEMs were identified in the petal samples. There were 147, 51, 117, and 57 DEMs between S0 vs. S1, S1 vs. S2, S2 vs. S3, and S3 vs. S4 (Fig. 2a), with 109, 26, 66, and 30 upregulated and 38, 25, 53, and 27 downregulated DEMs, respectively (Fig. 2b). We used Pearson correlation analysis to create a heat map of the correlations among the DEMs. The differential metabolite chord diagrams are shown in Fig. 2c and Supplementary Fig. 1. We observed a good correlation among the DEMs. K-means clustering analysis was performed to classify the expression patterns of the DEMs. A total of 12 sub-classes were obtained, and the number ascribed to each class was also recorded (Supplementary Fig. 2). Thermogram analysis showed that 97 metabolites were positively related to the formation of golden flowers (Fig. 2d). Of these, 79 metabolites were flavonols, including 40 quercetin-related glycosides and 27 kaempferol-related glycosides. In addition, 45 metabolites were negatively related to the formation of golden flowers, including eight proanthocyanidins and four anthocyanins (Fig. 2e). These results indicated that the accumulation of flavonol glycosides may be crucial to the formation of the golden flowers in *C. nitidissima*.

### Transcriptome analysis

Transcriptome sequencing was performed on the same 15 samples used for metabolome analysis with an Illumina HiSeq platform. After quality filtering, a total of 112.88 Gb clean reads were obtained. The clean data of all samples was not less than 6.37 Gb, with an average of 7.53 Gb. The proportion of the Q30 base was ≥ 92.39%, and the GC content reached 44.30–44.69% (Supplementary Table 1). A total of 122,201 unigenes were obtained after sequence assembly. The average length of the unigenes was 2219 base pairs (bp), with an N50 value of 2699 bp. To annotate the functions of these unigenes, their sequences were submitted to seven functional databases (KEGG, NR, Swiss-Prot, GO, COG/KOG, Trembl, and Pfam). The number of annotated unigenes in these databases ranged from 66,946 to 107,850, corresponding to the annotation percentages of 54.78–88.26% (Supplementary Table 1). Among them, 21,920 (20.14%) unigenes showed a high similarity with corresponding sequences in *Vitis vinifera* and 4892 unigenes exhibited good matches with corresponding genes from *C. sinensis*, followed by *Quercus suber* (Supplementary Fig. 3).

To validate the reproducibility and reliability of the RNA-seq data, 14 flavonoid- and hormone-related genes were subjected to qRT-PCR. The qRT-PCR results were consistent with the RNA-seq data (Supplementary Fig. 4), indicating that the RNA-seq results were reliable.

### Analysis of differentially expressed genes (DEGs)

**Fig. 3:**
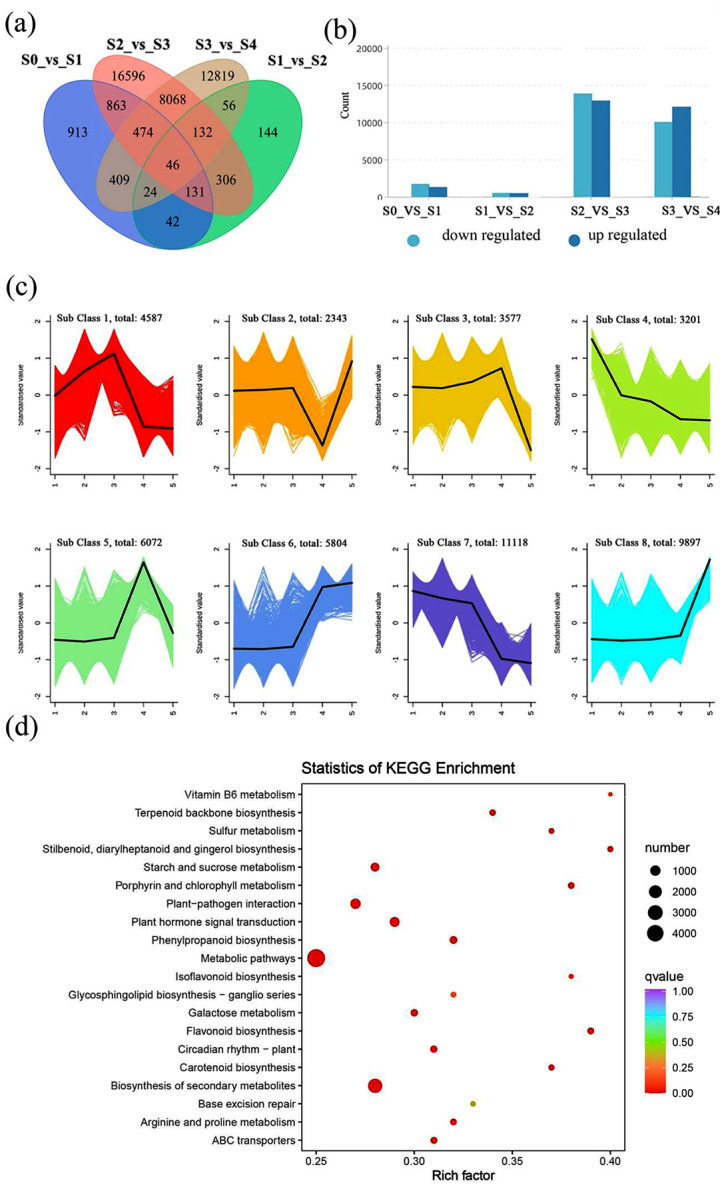
Transcriptome data analysis. (**a**) The Venn diagram depicts the common and unique number of (shared and unique) DEGs among the four groups of samples, respectively. (**b**) Up- and downregulated unigenes in different comparisons. The horizontal coordinate represents the number of DEGs, and the vertical coordinate represents different flowering periods. (**c**) Trend analysis of all DEGs performed using the K-means clustering analysis employing the z-score of the ion intensities. The horizontal coordinate represents the five developmental stages, and the vertical coordinate represents the centralized and standardized representation. (**d**) KEGG pathway classification of DEGs, Rich factor refers to the ratio between the Sample number of differential genes enriched in this pathway and the Background number of all genes annotated to this pathway. The greater the Rich factor, the greater the degree of enrichment. The smaller the Qvalue, the more significant the enrichment

Correlation analysis of the transcriptome samples was performed based on the expression levels of the DEGs. The results showed good reproducibility between samples (Supplementary Fig. 5). DEGs were screened using the thresholds of |log2 Fold Change| ≥ 1 and FDR < 0.05. A total of 46,637 DEGs were identified using the DESeq2/edgeR package. Moreover, 2902, 881, 26,616, and 22,028 DEGs were identified between S0 vs. S1, S1 vs. S2, S2 vs. S3, and S3 vs. S4 (Fig. 3a), with 1234, 423, 12,833, and 12,035 upregulated and 1668, 458, 13,783, and 9993 downregulated genes, respectively (Fig. 3b). K-means clustering analysis was used to classify the expression patterns of DEGs, and eight sub-classes were identified (Fig. 3c). Of these, sub-class 6 positively and sub-classes 4 and 7 negatively correlated with the flavonol accumulation in *C. nitidissima* (Fig. 3c). Furthermore, KEGG enrichment analysis revealed “Metabolic pathways,” “Biosynthesis of secondary metabolites,” “Plant hormone signal transduction,” and “Plant-pathogen interaction” to be the top four enriched pathways (Fig. 3d).

**Fig. 4:**
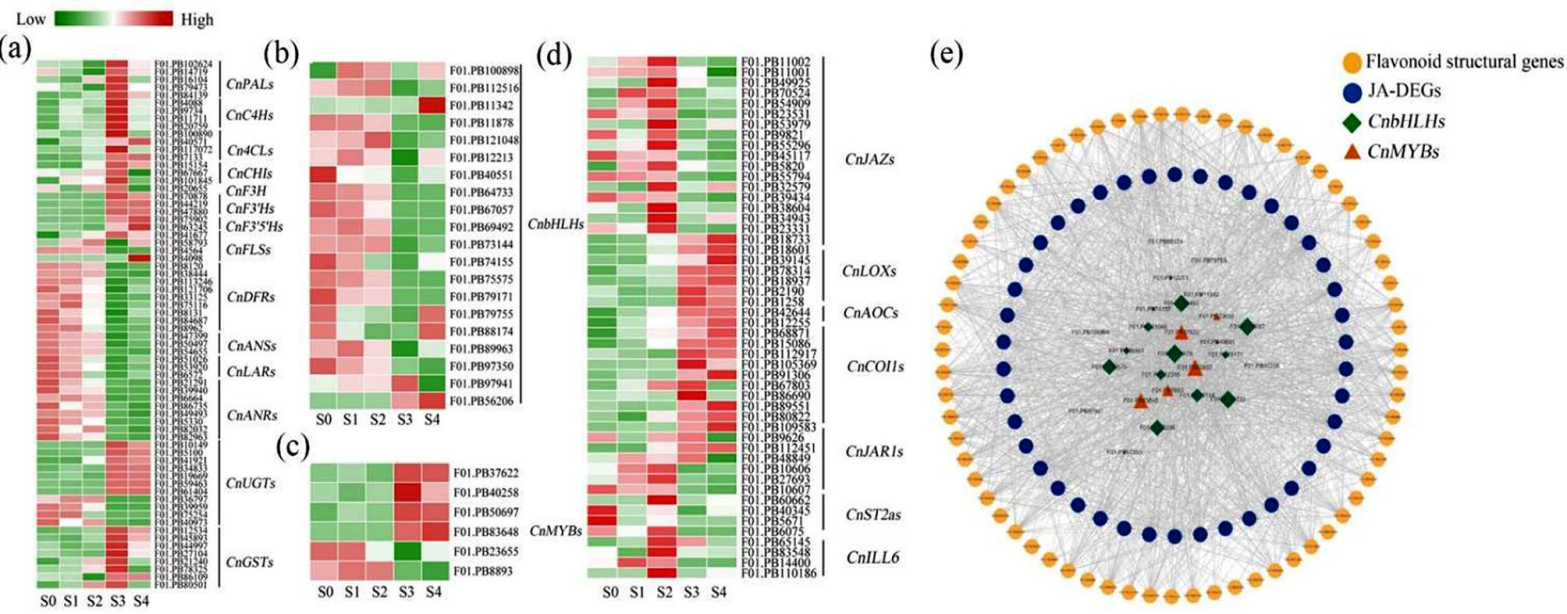
Heat maps and network correlation analysis. Heat maps of flavonoid (**a**), bHLH (**b**), MYB (**c**), and JA-related genes (**d**), green indicates low expression and red indicates high expression. (**e**) Network correlation analysis of *bHLHs*, *MYBs*, JA- and flavonoid-related genes. Retained only strong (|cor| > 0.8) and statistically significant (*p* < 0.05) associations

We detected many DEGs involved in the flavonoid biosynthesis pathway (Fig. 4a), including the biosynthesis genes *PAL*, *C4H*, *4CL*, *CHI*, *F3H*, *FLS*, *DFR*, and *ANS*. Many *MYB* and *bHLH* genes were obtained (Supplementary Fig. 6), of which 20 *bHLH* (Fig. 4b) and 6 *MYB* (Fig. 4c) genes were annotated in the flavonoid pathway. In addition, several hormone-related genes were also screened (Fig. 4d). These included JA biosynthesis-related genes *LOXs*, *AOCs*, and *JAR1s*; JA receptor-related gene *COI1s*; JA degradation-related genes *IAR3/ILL6s* and *ST2as*; and JA signaling-related genes *JAZs*. Our results indicated that flavonoid-related, JA-related, *MYB*, and *bHLH* genes might play key roles in flavonol biosynthesis in *C. nitidissima* petals. Network correlation analysis showed that JA-related, *MYB*, and *bHLH* genes were closely related to flavonoid biosynthesis genes (Fig. 4e).

### Proteome sequencing analysis

**Fig. 5:**
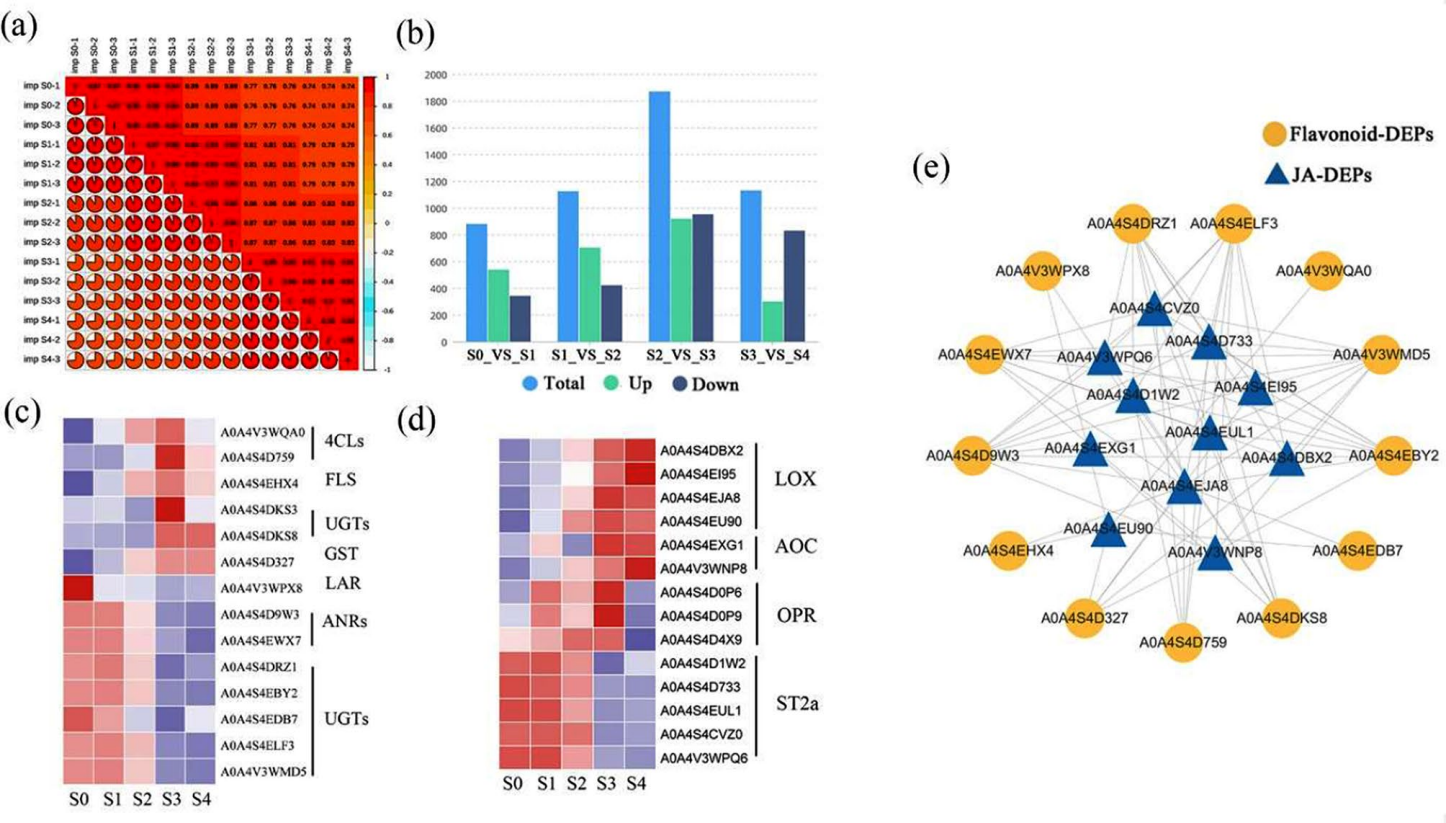
Analysis of proteome data. (**a**) Correlation heat map of proteome data samples. Pearson’s Correlation Coefficient (r) was used as an evaluation index of biological repeat correlation. The closer (R2) to 1, the stronger the correlation between the two duplicate samples. (**b**) Histogram of distribution of differential proteins in each period. Heat maps of flavonoid (**c**), and JA-related genes (**d**), purple indicates low expression and red indicates high expression. (**e**) Network correlation analysis of JA- and flavonoid-related proteins. Retained only strong (|cor| > 0.8) and statistically significant (*p* < 0.05) associations

Proteome sequencing was performed on the same 15 petal samples used for metabolome and transcriptome analyses. A high Pearson’s correlation coefficient for each group indicated that our data was reliable and could be used for subsequent analyses (Fig. 5a). In total, 27,173 peptides were detected, and 6642 proteins were confidently identified. For individual samples, 17,255 to 23,405 peptides and 5319 to 6082 proteins were identified (Supplementary Table 3). A total of 3842 differentially expressed proteins (DEPs) were screened out using the thresholds of fold change ≥ 1.5, *P* < 0.05 for upregulated and fold change ≤ 1.5, *P* < 0.05 for downregulated proteins. Furthermore, 880, 1124, 1870, and 1130 DEPs were detected between S0 vs. S1, S1 vs. S2, S2 vs. S3, and S3 vs. S4, including 538, 703, 919, and 300 upregulated and 342, 421, 951, and 830 downregulated DEPs, respectively (Fig. 5b). KEGG enrichment analysis revealed that the DEPs primarily enriched in “Metabolic pathways” and “Biosynthesis of secondary metabolites” (Supplementary Fig. 7).

### DEP analysis

During proteome analysis, several DEPs were found to participate in the flavonoid biosynthesis process, which was consistent with findings from the transcriptome data. These DEPs included two 4CLs, one FLS, one GST, one LAR, two ANRs, and seven UGTs (Fig. 5c). Moreover, many JA-related DEPs were detected (Fig. 5d), including JA biosynthesis proteins LOXs, OPRs, and AOCs and JA metabolism-related protein ST2as. Furthermore, network correlation analysis showed that JA-related and flavonoid biosynthesis-related proteins were closely related (Fig. 5e), consistent with the results of the transcriptome analysis.

### Flavonoid biosynthesis pathway in petals of *C. nitidissima*

**Fig. 6:**
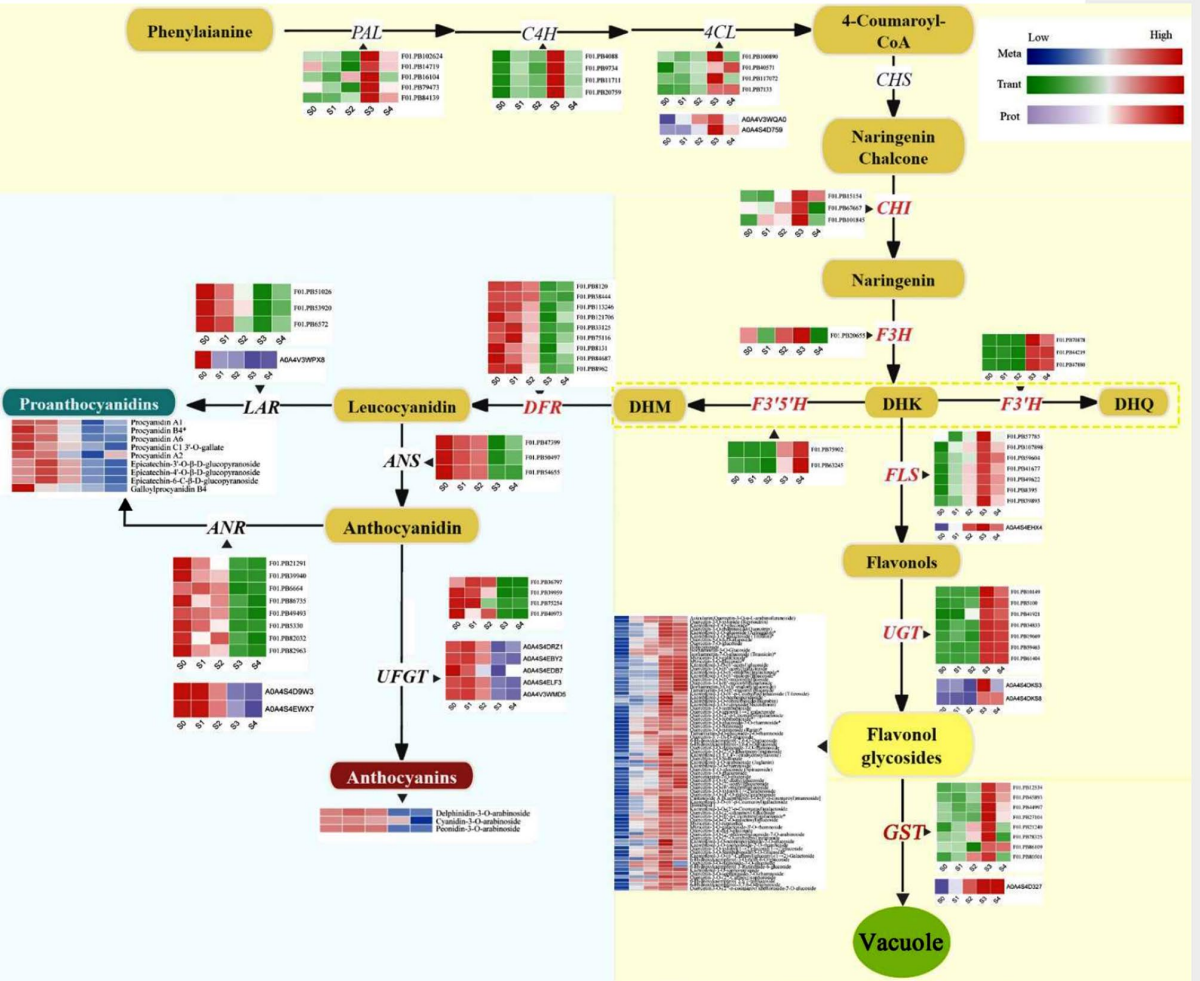
DEGs, DEPs and DEMs involved in the biosynthesis of the flavonoid pathway in *C. nitidissima*. PAL, phenylalanine ammonia lyase; C4H, cinnamate 4-hydroxylase; 4CL, 4-coumarate: CoA ligase; CHS, chalcone synthase; CHI, chalcone isomerase; F3H, flavanone 3-hydroxylase; F3’H, flavonoid 3′-hydroxylase; F3’5’H, flavonoid 3′5′-hydroxylase; FLS, flavonol synthase; UGT, flavonol 3-O-glucosyltransferase; DFR, dihydroflavonol 4-reductase; LAR, leucoanthocyanidin reductase; ANS, anthocyanidin synthase; ANR, anthocyanidin reductase; UFGT, flavonoid 3-O-glucosyltransferase; anthocyanidin 3-O-glucosyltransferase; GST, glutathione S-transferase

DEGs, DEPs, and DEMs were mapped to the flavonoid biosynthesis pathway using conjoint analysis (Fig. 6). Our results showed differential expressions of 15 key flavonoid biosynthesis-related genes or proteins across the five developmental stages of *C. nitidissima* flowers. Moreover, their expression patterns were consistent with the changes in flavonoid content (Fig. 6). The transcript abundances of six key structural genes, including *PAL* (five DEGs), *C4H* (four DEGs), *CHI* (three DEGs), *F3H* (one DEGs), *F3’H* (three DEGs), and *F3’5’H* (two DEGs) genes, were higher in the S3 and S4 (golden petal) stages than in other stages. Their expression levels in these stages were consistent with the changes in flavonol abundance and petal color. For 4CL, four DEGs and two DEPs were detected, and their expression patterns were consistent with the changes in flavonol accumulation. For FLS, the key enzyme in the flavonol biosynthesis, seven DEGs and one DEP were identified in the conjoint analysis. For UGT, which catalyzes flavonol glycosylation, seven DEGs and two DEPs were detected. For GST, which might be responsible for transferring flavonol glycosides to vacuoles, eight DEGs and one DEP were identified.

Furthermore, during the conversion of dihydroflavonols to anthocyanins and proanthocyanidins, we observed a downregulation of *DFRs* (nine DEGs), *ANSs* (three DEGs), *LARs* (three DEGs and one DEP), *ANRs* (eight DEGs and two DEPs), and *UGTs* (four DEGs and five DEPs). Their expression patterns contradicted FLS expression and flavonol accumulation but were consistent with the biosynthesis of anthocyanins and proanthocyanidins.

### Involvement of JA in the regulation of flavonol biosynthesis

**Fig. 7:**
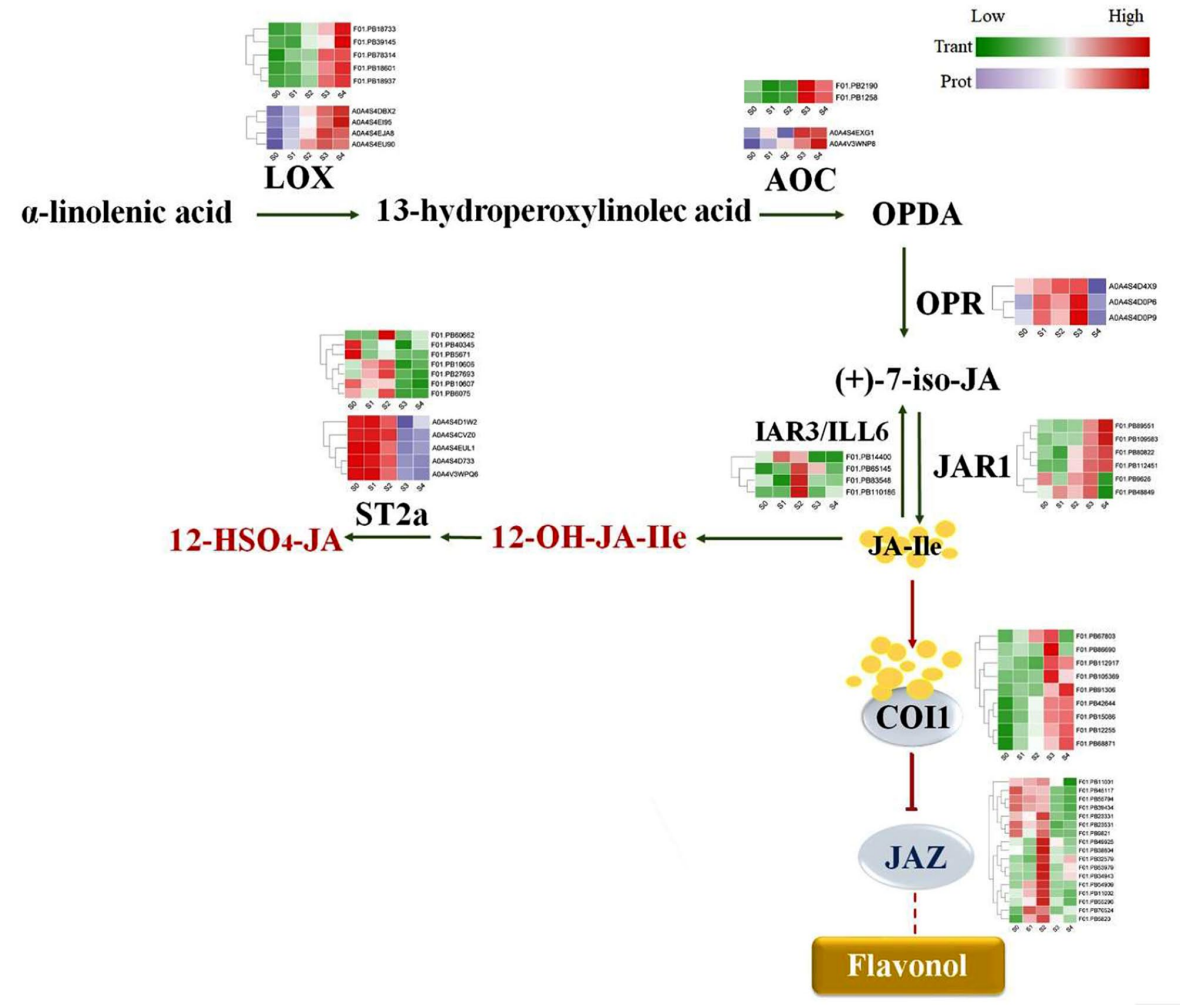
A model for the involvement of JA in the biosynthesis of flavonoids in *C. nitidissima*. LOX, enzymes lipoxygenase; AOC, allene oxide cyclase; OPR, OPDA reductase; IAR3 and ILL6, two JA amidohydrolases; JAR1, JA-amido synthetase; JA-Ile, (+)-7-iso-Jasmonoyl-L-isoleucine; ST2a, sulfotransferase 2a; COI1, F-box protein coronatine insensitive 1; JAZ, Jasmonate ZIM domain

As shown in Fig. 7, the expressions of JA biosynthesis-related genes, including *LOX*, *AOC*, and *OPR*, were higher in late developmental stages (S3 and/or S4) than in the early stages (S0, S1, and S2). Furthermore, IAR3/ILL6 and ST2a, which degrade JA-Ile and suppress JA signaling, were highly expressed in the early stages, while the JA receptor-related protein COI1 was upregulated in the late stages. Finally, contrary to the JA biosynthesis- and receptor-related genes, the expression of *JAZ*, the core regulator of JA signaling, decreased with flower development. These results suggested that JA was associated with flavonol biosynthesis in *C. nitidissima*.

### JA and CnFLS2 regulate flavonol biosynthesis

**Fig. 8:**
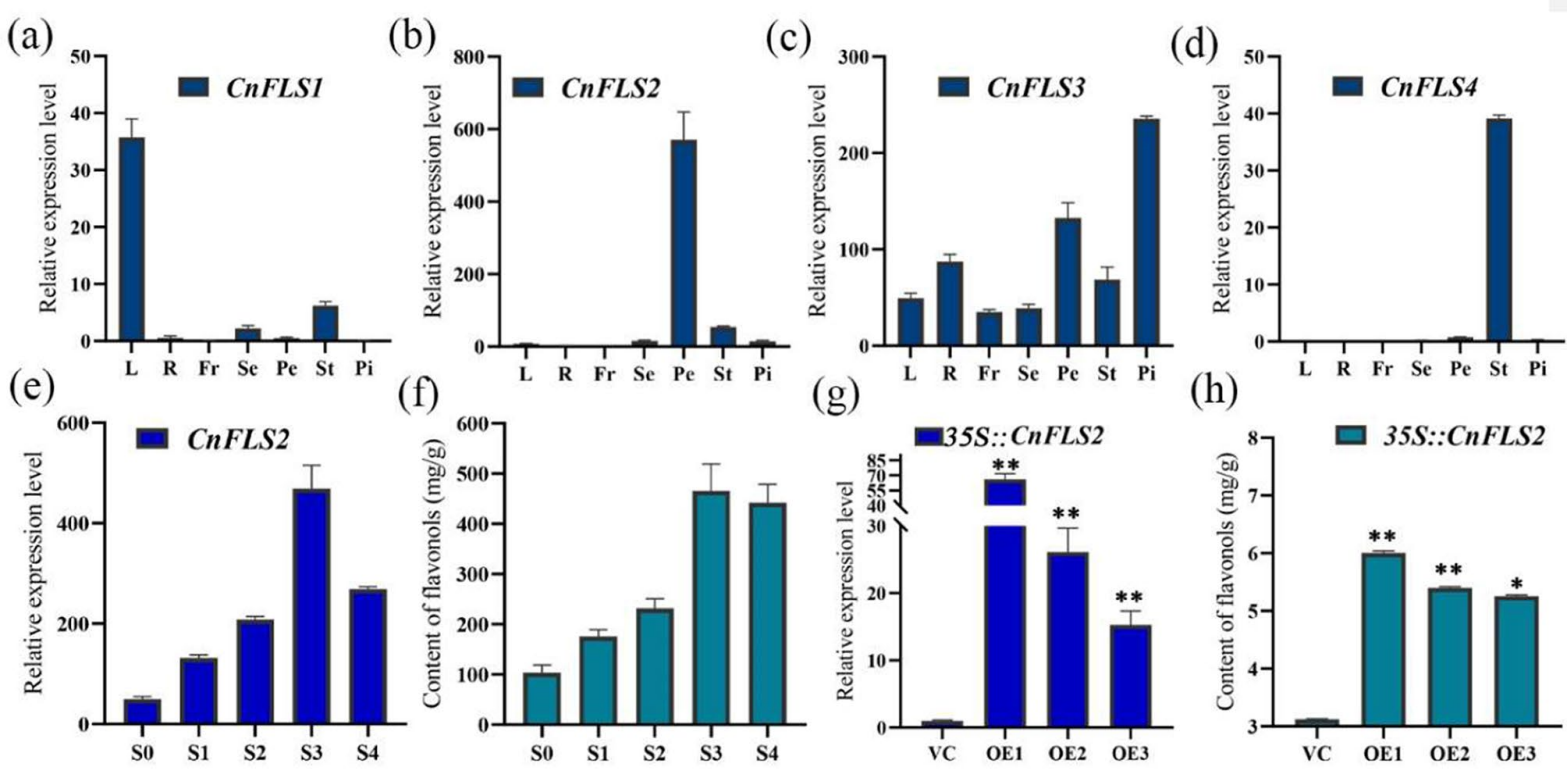
The expression analysis and transient overexpression of *CnFLS2* in *C. nitidissima*. Tissue-specific analysis of *CnFLS1* (**a**), *CnFLS2* (**b**), *CnFLS3* (**c**), *CnFLS4* (**d**); L means leaf, R means root, Fr means fruit, Se means sepal, Pe means petal, St means stamen, Pi means pistil. The expression of *CnFLS2* (**e**) and content of flavonol (**f**) at five developmental stages of petals. S0, early-bud stage; S1, mid-bud stage; S2, late-bud stage; S3, half-opening stage; S4, complete-opening stage. The expression of *CnFLS2* (**g**) and content of flavonol (**h**) analyses after transient overexpression treatment. VC, empty vector line; OE, overexpression line. Statistical significance was determined using Student’s t-test (*P < 0.05, **P < 0.01)

FLS is the key and rate-limiting enzyme in the flavonol biosynthesis pathway (Liu et al., 2021). We detected several *FLS* transcripts during transcriptome analysis, and sequence alignment revealed four *FLS* genes in *C. nitidissima*, named *CnFLS1*, *CnFLS2*, *CnFLS3*, and *CnFLS4* (Supplementary Fig. 8). Tissue-specific analysis showed that only *CnFLS2* was highly expressed in the petals (Fig. 8b). *CnFLS1* and *CnFLS4* were primarily expressed in the leaves and stamens (Fig. 8a, d) and *CnFLS3* was mainly expressed in the pistils (Fig. 8c). Transcriptome and metabolome analyses showed that *CnFLS2* expression in the petals at the five developmental stages positively correlated with the flavonol content (Fig. 8e, f).

Next, we transiently transformed *C. nitidissima* petals with a *CnFLS2* overexpression vector. The successfully transformed petals exhibited significantly higher *CnFLS2* expression (Fig. 8g) and enhanced flavonol accumulation (Fig. 8h). The results suggested that *CnFLS2* might be involved in flavonol biosynthesis in *C. nitidissima* petals.

**Fig. 9:**
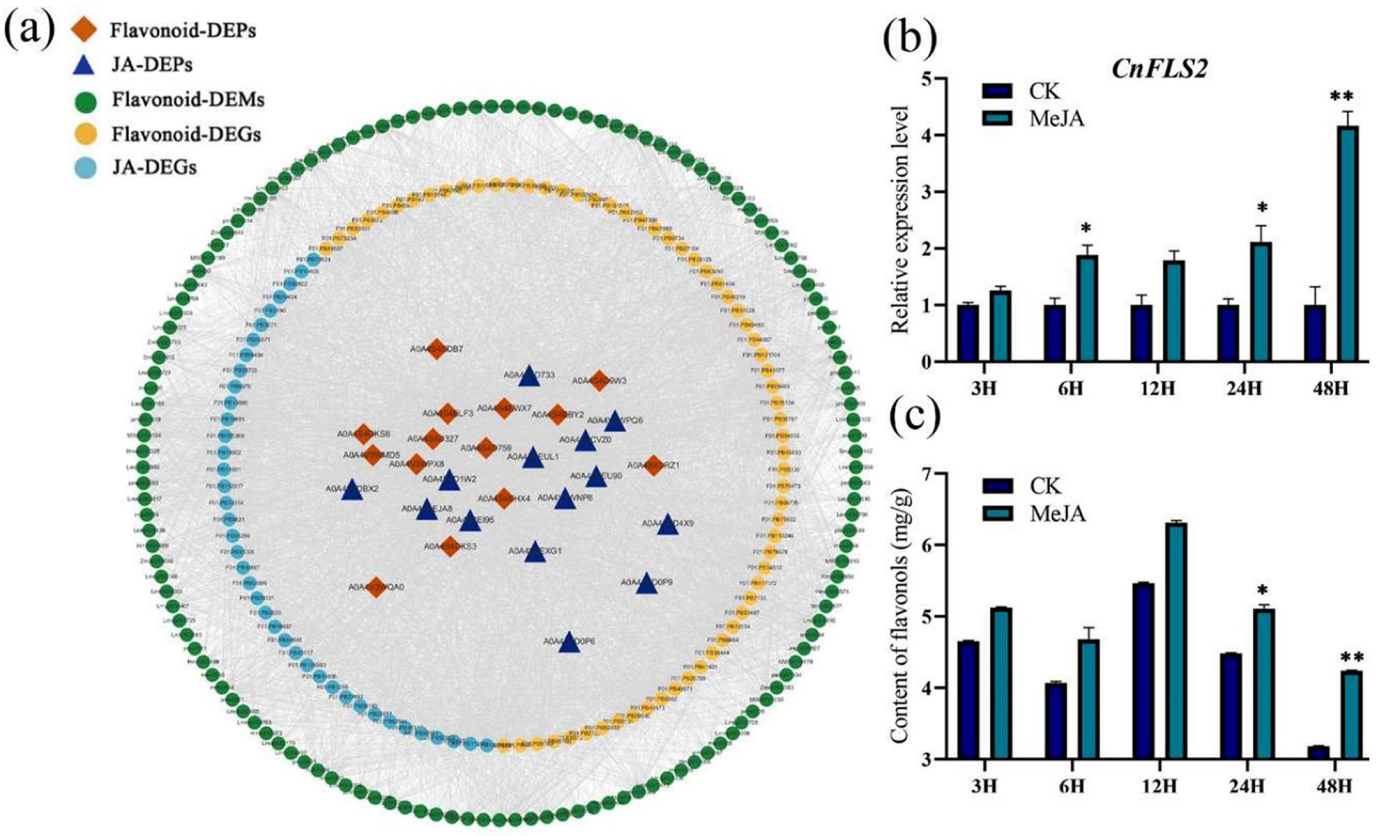
The conjoint analysis (**a**) of DEMs, DEGs, and DEPs, and expression of *CnFLS2* (**b**) and content of flavonol (**c**) after MeJA treatment. Statistical significance was determined using Student’s t-test (*P < 0.05, **P < 0.01)

The conjoint analysis of the detected DEMs, DEGs, and DEPs indicated strong correlations between JA-related pathways and flavonol biosynthesis (Fig. 9a). Furthermore, the correlation analysis of the transcriptome (Fig. 4e) showed that *CnFLS2* expression strongly correlated with 16 JA-related transcripts. More specifically, *CnFLS2* expression positively correlated with JA biosynthesis- and receptor-receptor genes *LOX*, *JAR1*, and *COI1* and negatively correlated with JA signaling- and degradation-related genes *JAZ* and *ST2a* (Fig. 4e). The correlation analysis of the proteome (Fig. 5e) showed a strong positive correlation between FLS2 (A0A4S4EHX4) and two LOXs (A0A4S4EJA8 and A0A4S4EU90).

Furthermore, *C. nitidissima* petals were subjected to exogenous MeJA treatment to further explores the relationships among the flavonols, FLS2 expression, and the JA pathway. We found that exogenous MeJA treatment promoted *CnFLS2* expression (Fig. 9b) and flavonol accumulation (Fig. 9c) in the petals. These results suggested that JA might regulate *CnFLS2* expression and impact flavonol synthesis in *C. nitidissima* petals.

## Discussion

### Flavonol biosynthesis during golden petal formation in *C. nitidissima*

Flower color is a key ornamental trait of ornamental plants and an important indicator of the quality and value of flowers. Camellia varieties with yellow flowers are scarce [31], and *C. nitidissima* is a rare and prized species with golden-yellow flowers. In addition, it is a valuable resource for research on yellow camellia flower formation and yellow camellia breeding. In this study, we conducted a metabolome analysis of the *C. nitidissima* petals at five developmental stages of the flowers (Fig. 1a). A total of 323 flavonoid metabolites were detected, with flavonols being the most abundant flavonoids (*n* = 114, 35.29%) (Fig. 1c). Compared with other *Camellia* species, the golden petals of *C. nitidissima* exhibit a higher accumulation of flavonol glycosides, including quercetin-7-O-glucoside, quercetin-3-O-glucoside, and quercetin-3-O-rutinoside [30, 31]. In our samples, we detected 79 flavonol glycosides, including quercetin-related glycosides and kaempferol-related glycosides (Fig. 2d), that positively correlated with the golden petal formation. These results suggested that quercetin and kaempferol glycosides are the main flavonoid metabolites in the golden petals of *C. nitidissima*.

The biosynthetic pathways of plant flavonoids have been previously elucidated [3, 8, 33]. Structural genes, such as *PAL*, *CHI*, *CHS*, *FLS*, *DFR*, *ANS*, *LAR*, and *ANR*, are related with the generation of flavonols, anthocyanins, and proanthocyanidins [34–36]. In the current study, transcriptome and proteome sequencing was implemented to explore the regulatory pathways underlying flavonol accumulation. We detected high expressions of flavonol biosynthesis-related genes *PAL*, *C4H*, *4CL*, *CHI*, *F3H*, *F3’H*, *F3’5’H*, *UGT*, *GST*, and *FLS* in the golden petal phase (Fig. 6), consistent with the elevated accumulation of flavonol glycosides. *FLS* and *DFR* are key structural genes that mediate the entry of flavonoids into different synthetic branches [3, 37, 38]. Studies have also shown that DFR and FLS compete for common dihydroflavonol substrates, and these proteins exhibit inhibitory effects on each other [18, 39]. Heterologous expression of *RrDFR1* and *PhDFR* in tobacco has been reported to inhibit the endogenous *NtFLS* expression and promote the biosynthesis of anthocyanins [20]. In the present study, *DRF* was highly expressed during the early flower developmental stages (S0–S2), diverting the flavonoids toward the anthocyanins and proanthocyanidins biosynthesis pathway. When petals entered the golden stage (S3–S4), *DFR* was downregulated, and *FLS* was highly expressed, promoting flavonol generation. Thus, the upregulation of flavonol biosynthesis-related genes, especially *FLS*, and the downregulation of the genes related to anthocyanin and proanthocyanidin biosynthesis leads to substantial biosynthesis of flavonol glycosides during golden flower formation.

MYB and bHLH transcription factors are vital regulators of flavonoid metabolism [40–42]. For instance, GtMYBP3 and GtMYBP4 are known to regulate genes involved in the early biosynthesis of flavonoids and promote flavonol biosynthesis [43]. The bHLH transcription factor AcB2 has been shown to interact with AcMYB1 and enhance anthocyanin biosynthesis in onion [44]. In the current study, network correlation analysis showed that 16 *bHLH* and 5 *MYB* genes were closely related to flavonoids, hormone-related genes, and flavonoid metabolites (Fig. 4e), indicating that these genes might be the key regulators of the flavonoid pathway in *C. nitidissima*.

### *CnFLS2* is crucial to flavonol biosynthesis during golden petal formation in *C. nitidissima*

*FLS* is the key structural and rate-limiting gene of flavonol biosynthesis in plants [3]. *FLS* overexpression has been shown to increase the biosynthesis of flavonols [45, 46]. Three *FLS* genes, *CsFLSa/b/c*, exist in the tea plant (*C. sinensis*). Heterologous overexpression of *CsFLSa/b/c* in tobacco has been shown to promote flavonol accumulation and the reduction of anthocyanidin levels in the petals [18]. In *A. thaliana*, *AtFLS1* overexpression changes the seed coat color light brown, while the *fls1-3* mutant exhibits enhanced anthocyanidin accumulation [47]. In the current study, the *CnFLS2* expression patterns during petal development positively correlated with the flavonol content (Fig. 8e, f). These results showed that *CnFLS2* might play a key role in the flavonol biosynthesis in *C. nitidissima* petals. And instantaneous expression of *CnFLS2* in petals promoted the expression of *CnFLS2* (Fig. 8g) and increased the content of flavonol (Fig. 8h). These results further suggested that *CnFLS2* might be crucial to flavonol synthesis in *C. nitidissima* petals.

### JA is involved in the regulation of flavonol biosynthesis during golden petal formation

Hormones are crucial for several plant growth and development processes, including flavonoid metabolism [33, 48]. Some phytohormones include auxin [49], JA [50], GA [51], BR [52], strigolactone [53], ethylene [54], and abscisic acid [55]. JA positively regulates the biosynthesis of various flavonoids, including anthocyanins [56], proanthocyanidins [57, 58], flavonols, and flavones [59]. In the current study, we detected many JA biosynthesis- and signaling-related genes (Fig. 4d) and proteins (Fig. 5d) in *C. nitidissima* petals. Previously, exogenous MeJA treatment has been shown to promote the flavonol biosynthesis-related genes *FLS*, *F3H*, *CHS*, and *CHI* and transcription factor *MYB81* in *Gynostemma pentaphyllum* [60]. In our samples, the expression patterns of JA biosynthesis-related genes (Fig. 4d) were consistent with flavonol contents (Fig. 2d) and the expression patterns of flavonol biosynthesis-related genes (Fig. 4a). Moreover, opposite trends were observed from the genes that negatively regulate the JA biosynthesis pathway. These results indicated that JA might positively regulate flavonol biosynthesis in *C. nitidissima* petals. Previously, exogenous MeJA treatment has been found to increase the content of several flavonols, such as myricetin and quercetin, and PAL activity [25]. In the current study, MeJA treatments promoted *CnFLS2* expression (Fig. 9b) and flavonol accumulation (Fig. 9c). These results suggested that the JA-*CnFLS2* module regulated flavonol biosynthesis in petals of *C. nitidissima* petals. However, the regulatory effects of JA on *CnFLS2* expression and flavonol biosynthesis need to be further explored.

## Conclusion

This study provided insights into the regulatory mechanism underlying flavonol biosynthesis in *C. nitidissima* petals via an integrative multi-omics analysis. A total of 323 flavonoids were detected in the petals during the five flower developmental stages, with flavonols being the most abundant among them (*n* = 114, 35.29%). Of these, quercetin and kaempferol glycosides were the most highly expressed in the golden petals. The transcriptome and proteome sequencing suggested an elevation in the expressions of flavonol biosynthesis-related genes and proteins in the golden petal stage. Correlation analysis showed that *CnFLS2* expression in the petals positively correlated with the flavonol content across all flower developmental stages. Furthermore, *CnFLS2* overexpression in the petals was found to increase flavonol content. In addition, the JA-related pathways positively correlated with flavonol biosynthesis, and exogenous MeJA treatment induced *CnFLS2* expression and flavonol accumulation.

## Materials and methods

### Plant materials

*C. nitidissima* plants were obtained from the Camellia Germplasm Resources of the Institute of Subtropical Forestry, Chinese Academy of Forestry (Qianjia village, Hangzhou city, Zhejiang province), where they were grown in the field. All plants were about 15 years old. Petals were collected at five different flower development stages in February 2021: Early-bud (S0), mid-bud (S1), late-bud (S2), half-opening (S3), and complete-opening stages (S4). The petals were then immediately frozen in liquid nitrogen and stored at − 80 °C for subsequent analysis.

### Metabolic analysis

The obtained samples were freeze-dried and powdered. Then, 100 mg of each lyophilized powder was mixed with 1.2 mL of 70% methanol. The extracts were filtrated (SCAA-104, 0.22 μm pore size; ANPEL, Shanghai, China, http://www.anpel.com.cn/). The metabolites in the filtrates were quantified using ultra-high performance liquid chromatography (UPLC)-electrospray ionization (ESI)-tandem mass spectrometry (MS/MS). For UPLC, the Agilent SB-C18 column (1.8 μm, 2.1 mm × 100 mm) was used. The mobile phase included solvent A, comprising pure water mixed with 0.1% formic acid, and solvent B, comprising acetonitrile mixed with 0.1% formic acid. Sample measurements were performed with a gradient program that employed the starting conditions of 95% A and 5% B. Within 9 min, a linear gradient to 5% A and 95% B was programmed and a composition of 5% A and 95% B was kept for 1 min, Subsequently, a composition of 95% A, and 5% B was adjusted within 1.1 min and kept for 2.9 min.

Qualitative metabolite analysis was performed based on the self-built database MWDB (Metware Biotechnology Co., Ltd., Wuhan, China) and secondary spectral information. Each metabolite was quantified via multiple reaction monitoring analysis using triple four-stage mass spectrometry (MS). Unsupervised principal component analysis (PCA) and orthogonal partial least squares discriminant analysis (OPLS-DA) were used to analyze the metabolites. Significantly regulated metabolites between groups were identified using the criteria of variable importance in projection (VIP) ≥ 1. Metabolites with a fold change of ≥ 2 or ≤ 0.5 based on the OPLS-DA results were identified as significantly differential metabolites. Hierarchical clustering analysis of the metabolites in different samples was performed using the R software (www.r-project.org/). The differential metabolites were subsequently subjected to Kyoto Encyclopedia of Genes and Genomes (KEGG) analysis.

### RNA sequencing (RNA-seq) analysis

Total RNA was extracted from *C. nitidissima* flowers using an RNA Prep Pure kit for plants (Tiangen, Beijing, China). A total of 1 µg RNA per sample was used for RNA sample preparation. The index-coded samples were clustered using the cBot Cluster Generation System using TruSeq PE Cluster Kit v3-cBot-HS (Illumina). After cluster generation, the library preparations were sequenced on the Illumina HiSeq platform.

Use fastp v 0.19.3 to filter the original data, when any sequencing reads with > 50% low-quality (Q ≤ 20) bases were excluded. Transcriptome assembly was prepared using Trinity (v2.11.0). TransDecoder was used to identify candidate coding regions. The transcript sequences were generated by de novo RNA-Seq transcript assembly using Trinity. Gene expression levels were estimated using RNA-Seq by Expectation-Maximization (RSEM) [61]. The expression abundance of the corresponding unigenes was calculated using the Fragments per Kilobase of Transcriptome per Million Mapped Reads (FPKM) method. Gene function was annotated based on the following databases: KEGG, euKaryotic Ortholog Groups/Clusters of Orthologous Groups of Proteins (KOG/COG), Gene Ontology (GO), NCBI non-redundant protein sequences (Nr), and Trembl (a variety of new documentation files and the creation of TrEMBL).

DESeq2 [62] was used to analyze differential expressions between two groups. P-values were corrected using Benjamini and Hochberg’s method. The false discovery rate (FDR) was corrected using the posterior probability values. FDR < 0.05 and |log_2_ (foldchange)| ≥ 1 were regarded as the thresholds for significantly differential expression.

### Protein sample preparation and MS detection and data analysis

The protein fraction in the solution was precipitated using acetone. Each ground flower tissue was mixed with four volumes of lysis buffer (8 M urea, 100 mM Tris-Cl, and 10 mM dithiothreitol) and incubated at 37 °C for 1 h. ​Subsequently, 40 mM iodoacetamide was added to the mixture. The Bradford method was used to determine protein concentration in each sample. After protein quantification, 50 µg of the protein samples were resolved by sodium dodecyl sulfate-polyacrylamide gel electrophoresis (SDS-PAGE), and the protein bands were stained using Kemas Brilliant Blue. The extracted proteins were reduced, alkylated, and digested with trypsin. The obtained peptides were desalted using a Sep-Pak C18 column and vacuum-dried.

The MS data were acquired using a Q Exactive HF-X mass spectrometer in tandem with an EASY-nLC1200 liquid-phase liquid chromatography system. The DIA-NN software was used to establish a spectrum library based on the protein sequence database of *C. sinensis* var. *sinensis* in the UniProt database. The proteins were identified, and quantitative information was extracted. The test results were screened using a threshold of 1% FDR. The quantification intensity information obtained from DIA analysis was used for difference comparison, with t-test analysis after log2 transformation, data filling (imputation algorithm in Perseus software), and data normalization. The thresholds of fold change ≥ 1.5, *P* < 0.05 and fold change ≤ 1.5, *P* < 0.05 were set for upregulated and downregulated proteins, respectively.

### Interaction network analysis

An interaction network was established based on Pearson’s correlation coefficients, which were calculated using R (https://www.r-project.org/). Correlations with a coefficient of either *R* ≥ 0.8 or *R* ≤ − 0.8 and *P* ≤ 0.05 were retained. The relationships between candidate genes, including genes encoding transcription factors (TFs), structural proteins, and flavonoid components, were visualized using Cytoscape (v. 3.9.2).

### Quantitative real-time polymerase chain reaction (qRT-PCR) analysis

All gene-specific primers were designed using NCBI online. The RNA samples used for RNA-Seq were subjected to RT-qPCR. Each sample was analyzed thrice. *CnGAPDH* was used as the internal standard for normalization (Supplementary Table 4). Relative gene expression levels were analyzed using the 2^−∆∆Ct^ method. The qRT-PCR protocol was set as described previously [31].

### Transient *CnFLS2* overexpression in *C. nitidissima*

Branches with flowers were cultivated in water for 1 day. The petals of complete-opening stage were used to induce transient overexpression of *CnFLS2* using an Agrobacterium-mediated transformation system. Briefly, the target vectors (pCAMBIA1302*-CnFLS2* and pCAMBIA1302) were transferred into Agrobacterium strain GV3101. The transformed Agrobacterium cells were shaken till the culture reached an OD_600_ of 0.8. Then, the cultures were centrifuged at 5,000 rpm for 8 min. The precipitates were resuspended in the infiltration buffer (containing 10 mM MES, 10 mM MgCl_2_, and 100 µM acetosyringone (pH 5.7–5.8)) and injected into the petals. Then, the branches were cultivated at 24 °C in the dark for 24 h. Next, the branches were grown under a 16 h-light/8 h-dark cycle at 24 °C and 50% humidity (100 mmol m^− 2^ s^− 1^ with fluorescent lamps) for 48–50 h. Finally, the petals were collected and frozen in liquid nitrogen for subsequent analysis.

### MeJA treatment

Branches with flowers were used for MeJA treatment. Exogenous MeJA treatment was carried out as described previously [63]. In this method, the petals were treated with 250 µmol/L MeJA. The control groups were treated with H_2_O, the solution was dissolved in 1% ethanol. These solutions contained 0.01% Tween-20 to promote absorption. The treated petals were extracted at 3, 6, 12, 24, and 48 h and frozen in liquid nitrogen for subsequent analysis.

### Flavonol content determination

Total flavonols were extracted from plant tissue using a plant flavonol extraction kit (Comin, Suzhou, China). Took 0.1 g of the samples to be tested to measure the total flavonol content, added the extraction solution according to the instructions, homogenized, ultrasonicated for 30 min, centrifuged, and took the supernatant. Added the reaction solution containing sodium nitrite and aluminum salt to the supernatant. After reacting at room temperature for 20 min, added sodium hydroxide solution. Used water as a blank control. Read the absorbance values of the measurement tube and control tube at 510 nm, respectively, and recorded them as A measurement and A respectively. Rutin was used as a standard in order to establish the calibration curve. Calculated the total flavonol content according to the formula.

$$\Delta A = A \left( {determination} \right) - A\left( {control} \right)$$

$${C_{flavonol (mg/ml)}} = [(\Delta A + 0.01) \div 3.0544 \times V1] \div \left( {W \times V1 \div V2} \right)$$

V1: The volume of the sample added to the reaction system; V2: The volume of the extraction solution added; W: Sample mass, 0.1 g.

### Statistical analysis

Statistical analyses were conducted using the SPSS (v25) software (SPSS Inc., Chicago, IL, USA). All experiments were repeated with at least thrice. The data were analyzed statistically using Student’s t-test. The differences were considered statistically significant at *P* < 0.05 or *P* < 0.01.

## Electronic supplementary material

Below is the link to the electronic supplementary material.

Supplementary Material 1

## Data Availability

All data generated or analyzed during this study are included in this published article (and its supplementary information files). The sequenced raw reads generated in this study have been submitted to the National Center for Biotechnology Information (NCBI) with BioProject ID: PRJNA909942.
