# Supplementary material for: Multi-omics analysis revealed the mechanism underlying flavonol biosynthesis during petal color formation in Camellia Nitidissima
Source: BMC Plant Biol. 2024 Sep 9;24:847. doi: 10.1186/s12870-024-05332-w (PMC11382509; doi:10.1186/s12870-024-05332-w)
Supplement: Supplementary file 1 — Supplementary Material 1 [file 12870_2024_5332_MOESM1_ESM.docx]

Supplementary Table 1 Summary of sequencing reads

| Sample | Raw Reads | Clean Reads | Clean Base(G) | Error Rate(%) | Q20(%) | Q30(%) | GC Content(%) |
| --- | --- | --- | --- | --- | --- | --- | --- |
| S0-1 | 51558866 | 50260490 | 7.54 | 0.03 | 97.77 | 93.47 | 44.6 |
| S0-2 | 53371998 | 51958692 | 7.79 | 0.03 | 97.67 | 93.25 | 44.57 |
| S0-3 | 57139526 | 55471652 | 8.32 | 0.03 | 97.72 | 93.34 | 44.69 |
| S1-1 | 52856320 | 50911218 | 7.64 | 0.03 | 97.64 | 93.2 | 44.45 |
| S1-2 | 52820950 | 50671948 | 7.6 | 0.03 | 97.59 | 93.11 | 44.53 |
| S1-3 | 51083526 | 49699510 | 7.45 | 0.03 | 97.5 | 92.88 | 44.34 |
| S2-1 | 54971700 | 53472876 | 8.02 | 0.03 | 97.44 | 92.66 | 44.33 |
| S2-2 | 43632422 | 42460506 | 6.37 | 0.03 | 97.65 | 93.15 | 44.3 |
| S2-3 | 53306428 | 51463876 | 7.72 | 0.03 | 97.28 | 92.39 | 44.38 |
| S3-1 | 54887108 | 53289074 | 7.99 | 0.03 | 97.52 | 92.93 | 44.58 |
| S3-2 | 46633716 | 45337340 | 6.8 | 0.03 | 97.69 | 93.29 | 44.63 |
| S3-3 | 53378168 | 51776964 | 7.77 | 0.03 | 97.49 | 92.77 | 44.59 |
| S4-1 | 47828198 | 46536160 | 6.98 | 0.03 | 97.62 | 93.12 | 44.62 |
| S4-2 | 52115654 | 50280564 | 7.54 | 0.03 | 97.59 | 93.08 | 44.53 |
| S4-3 | 50420868 | 49031126 | 7.35 | 0.03 | 97.53 | 92.89 | 44.58 |

Supplementary Table 2. Summary of functional annotation result of unigenes

| Database | Number of Genes | Percentage (%) |
| --- | --- | --- |
| KEGG | 83956 | 68.7 |
| NR | 107850 | 88.26 |
| SwissProt | 82993 | 67.92 |
| Trembl | 107745 | 88.17 |
| KOG | 66946 | 54.78 |
| GO | 93471 | 76.49 |
| Pfam | 82971 | 67.9 |
| Annotated in at least one Database | 108956 | 89.16 |
| Total Unigenes | 122201 | 100 |

Supplementary Table 3 Summary of sequencing proteins

| Samples | Number of proteins | Number of peptides |
| --- | --- | --- |
| S0-1 | 6054 | 23405 |
| S0-2 | 6080 | 23347 |
| S0-3 | 6082 | 23327 |
| S1-1 | 6053 | 22971 |
| S1-2 | 6026 | 22862 |
| S1-3 | 6061 | 22905 |
| S2-1 | 5993 | 22320 |
| S2-2 | 5990 | 22286 |
| S2-3 | 5996 | 22353 |
| S3-1 | 5914 | 21809 |
| S3-2 | 5945 | 21823 |
| S3-3 | 5900 | 21676 |
| S4-1 | 5319 | 17255 |
| S4-2 | 5340 | 17458 |
| S4-3 | 5384 | 17543 |
| total | 6642 | 27173 |

**Supplementary Table 4.** Sequences of primers used for the qRT-PCR.

| **Target** | **Forward primer (5’ to 3’)** | **Reverse primer (5’ to 3’)** |
| --- | --- | --- |
| *GAPDH*  *F01.PB8395*  *F01.PB38444*  *F01.PB64884*  *F01.PB37622*  *F01.PB11878*  *F01.PB4314*  *F01.PB13418*  *F01.PB15154*  *F01.PB50497*  *F01.PB81668*  *F01.PB6729*  *F01.PB19669*  *F01.PB61152*  *F01.PB103282*  *F01.PB53386* | GGGAATCCTTGGTTACACTGAG  AGCAATCACCACCGTCAAAGG GTGTTACAGGAGGTTCTGGGT  CGTCACCCGAGACCACTTC  CTTACATCCGATCCCACGGC  CAAGAAGGGGTATTGGAATGG  AGGTTCAGGAGTTCAGTGGTC  GAGGAGGCGGAATGGATGAG  GTGCTTTGCCCACTTTCGTT  AAGAGCTCACTAGCATCGGC  GACGATATCTCAGGTGGCGG  CGTCGTCCTTGCCATCACTA  GCATCTAGGGCTTGTGAGGG  TCTGTTCCGAAGCTGGTTGG  ACCGATCACGATCCAACAGG  ACACTCCCAAGAAACACACACATCC | ACCCCATTCGTTGTCATACC  CTCTTAGACTCAGCATCCTTAGC CTGGGAATTGTGCTGCAAGG  GGTGCAAAAACAAGGTCCTG  TCTTCCCGCTATAAGCGACC  CACAAAGGACATGCAAACCAAG  GGCGTTTGTGAGTGGTGTC  CGAGTGGTGAAGCGTTGTTG  GATCATCTGGGTGCGGAACA  TCTCCTCCACAGGCTGGTTA  TGTGGCCAGACTCTACTCCA  GACTTGGAGCCAGTTTCCGA  GCTGCATCACGCAACTTCAT  TTTGGGCTCGGTTGGATTGA  GGTCCAATGTAACCCGTCGT  GGTTTTGAGATGGGGGTGTTGT |

Supplementary Fig. 1. The Pearson correlation analysis of differential expressed metabolites (DEMs)
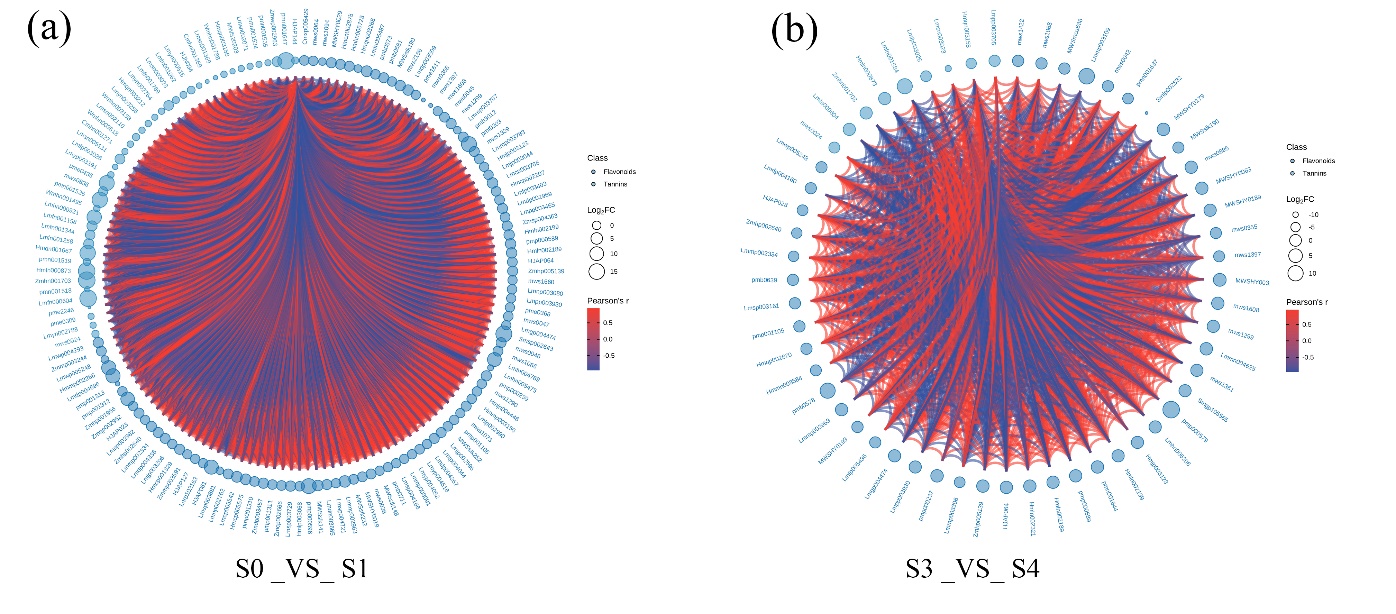
. The red line represents positive correlation, while the blue line represents negative correlation.


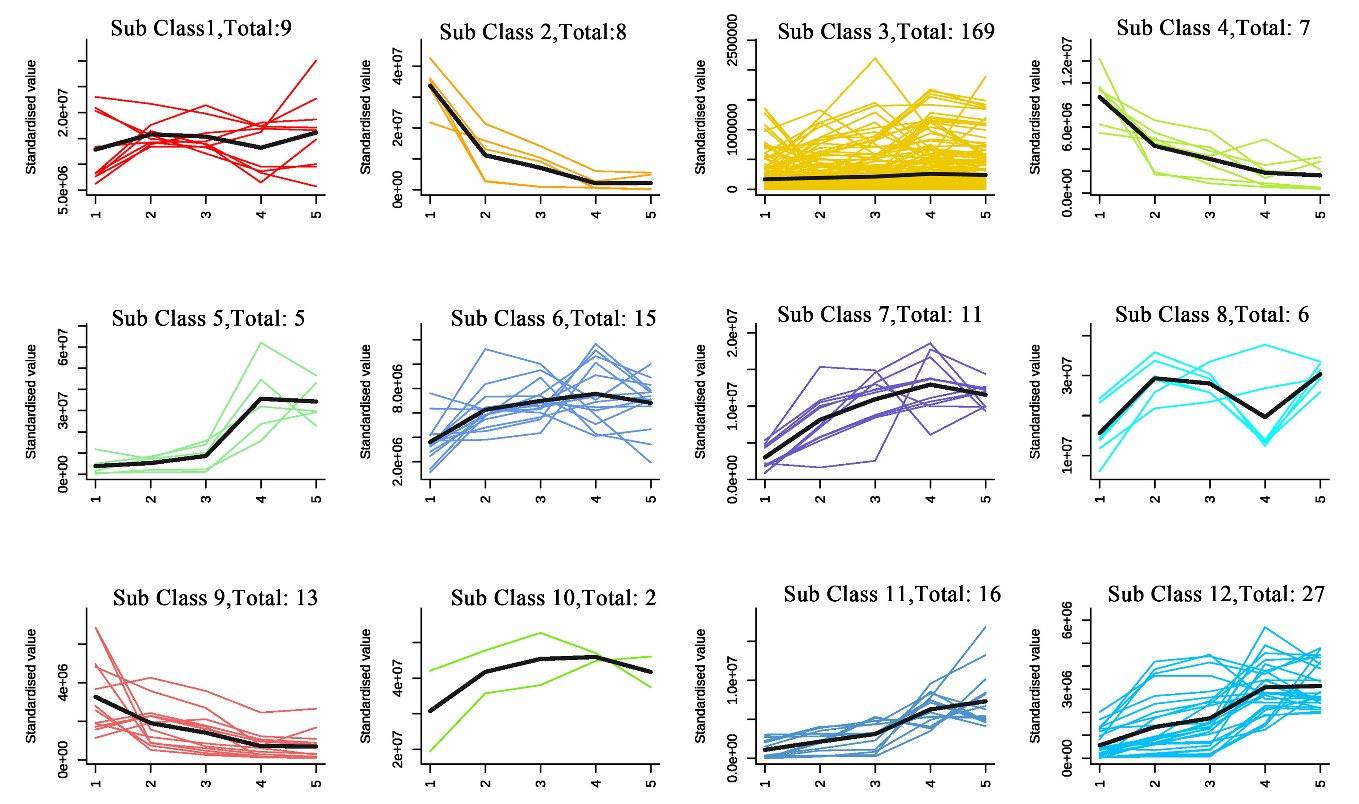


Supplementary Fig. 2. Expression trends of the identified DEMs in five developmental stages.


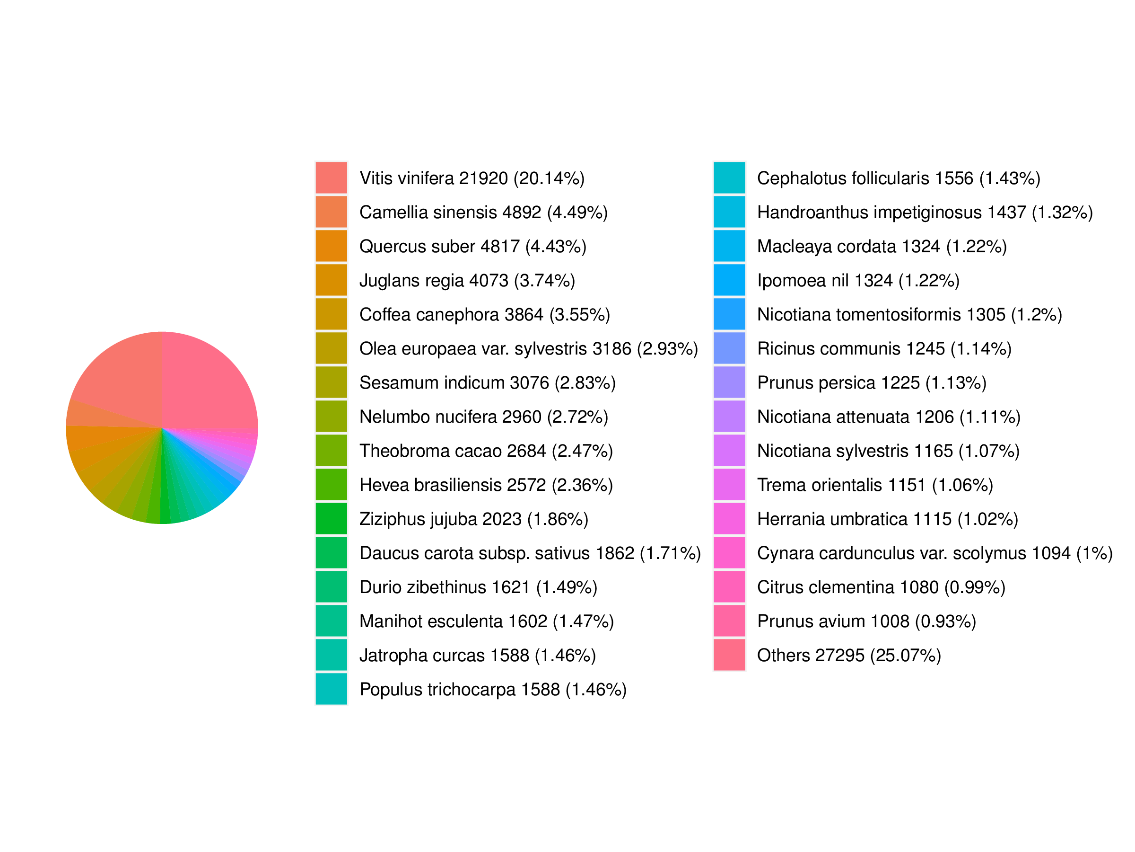


Supplementary Fig. 3. Annotated drawing with NR database.


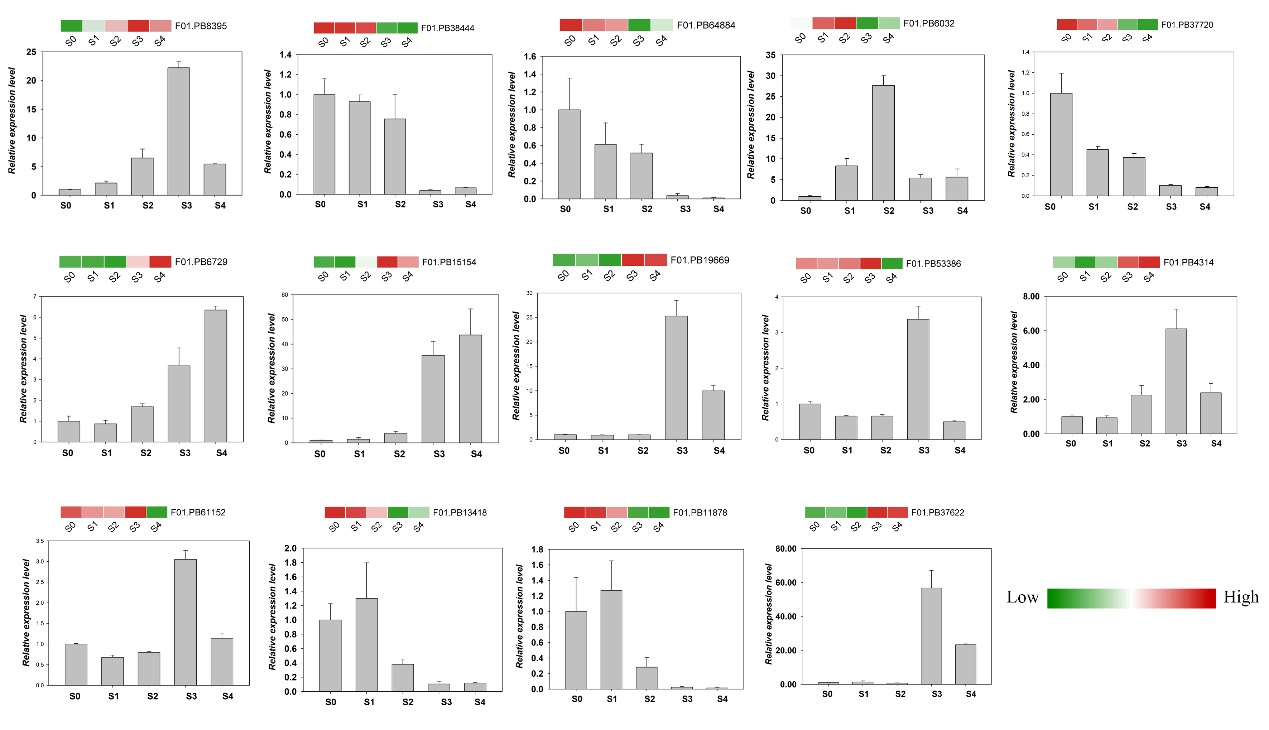


Supplementary Fig. 4. Validation of the expression of candidate genes by RT-PCR.


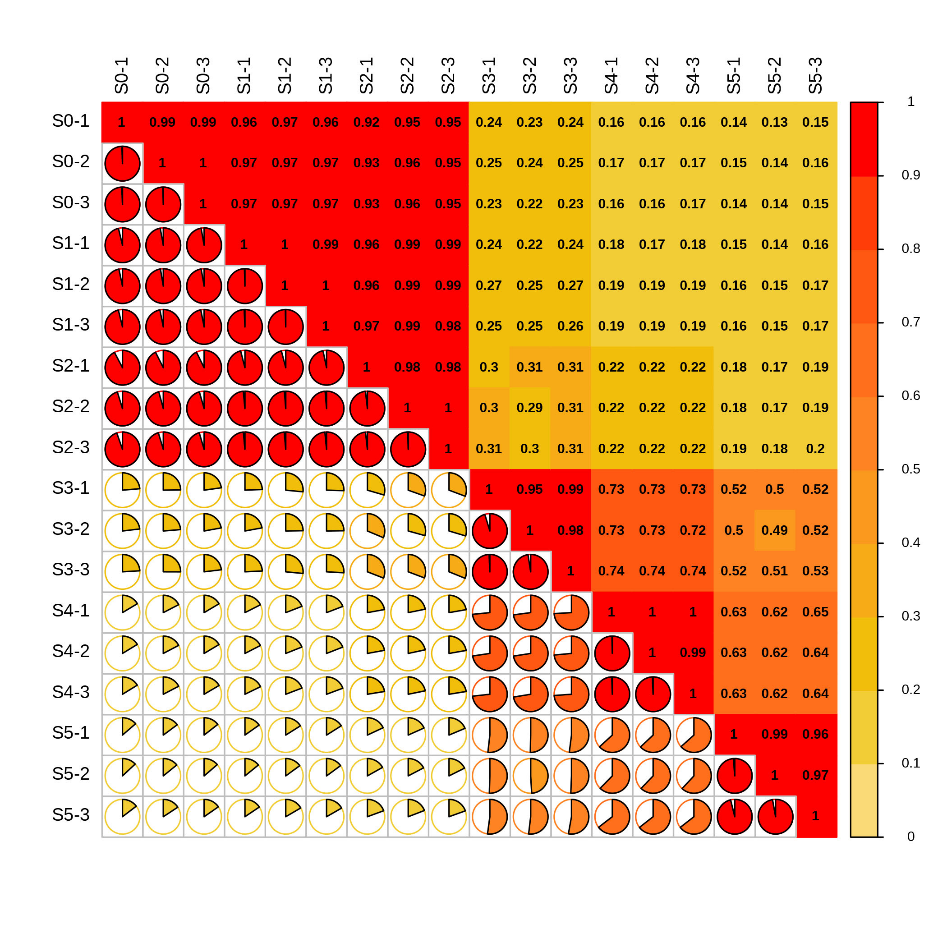


Supplementary Fig. 5. Transcriptome sample correlation analysis.


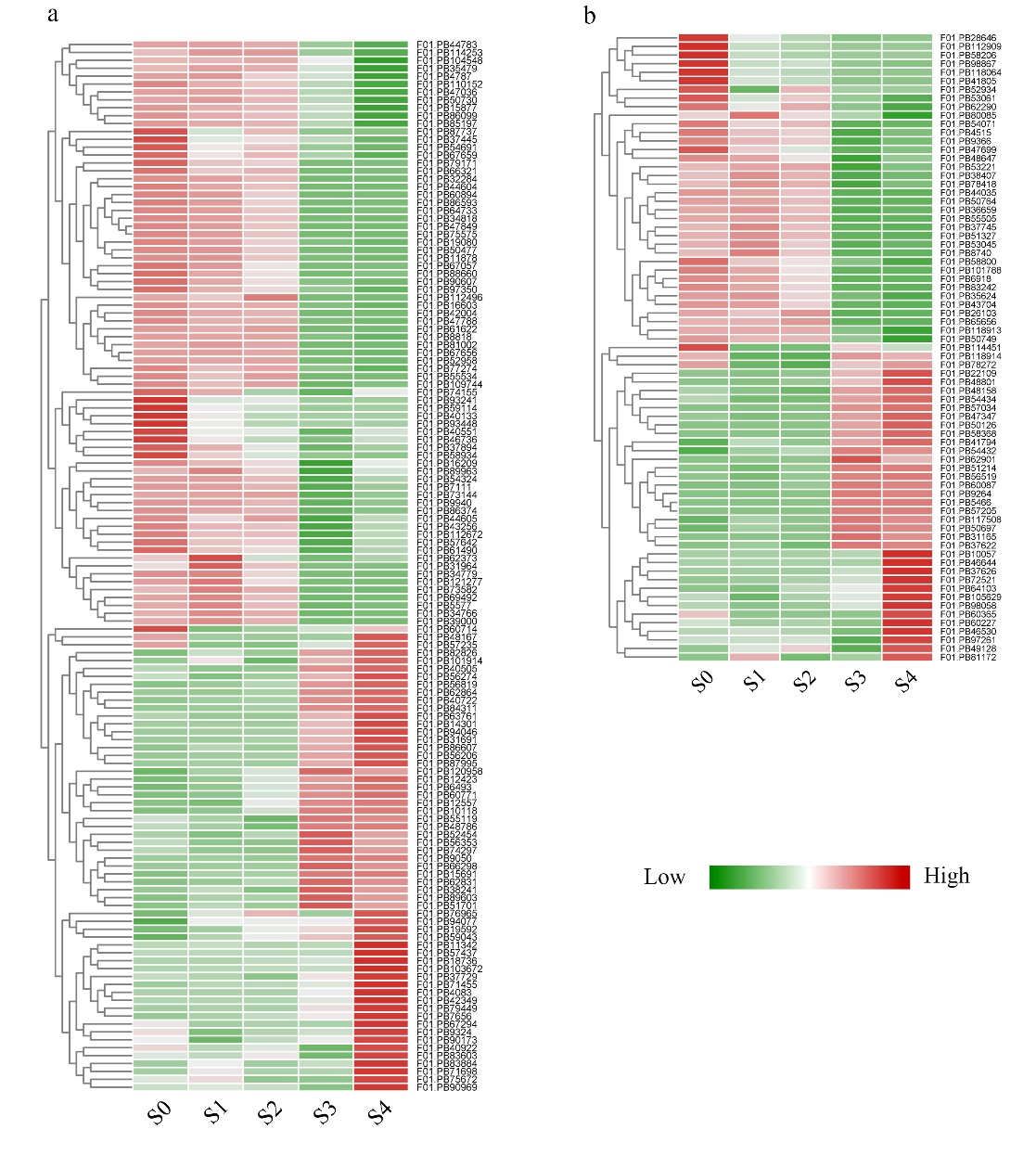
Supplementary Fig. 6. Heat map of *bHLH* (a) and *MYB* (b) genes.


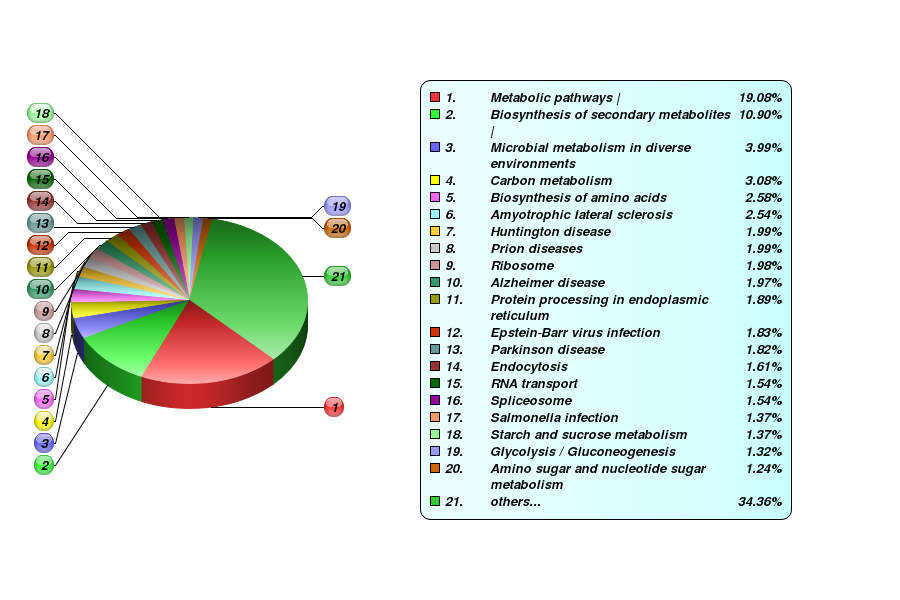


Supplementary Fig. 7. KEGG enrichment analysis of DEPs.

Supplementary Fig. 8. Heat map of *FLS* genes
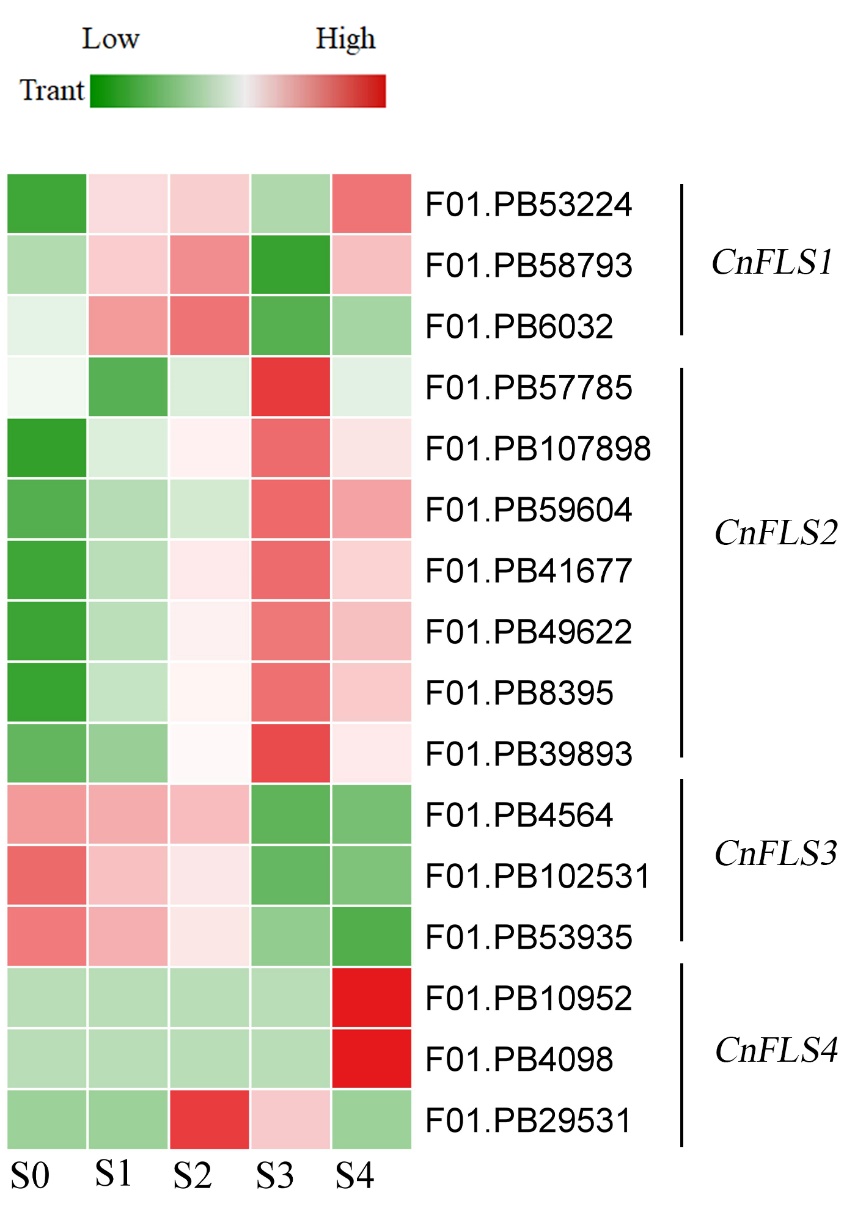
.
